# Myocardial Inflammation—Are We There Yet?

**DOI:** 10.1007/s12410-015-9320-6

**Published:** 2015-02-18

**Authors:** Simon Greulich, Vanessa M. Ferreira, Erica Dall’Armellina, Heiko Mahrholdt

**Affiliations:** 1 Division of Cardiology, Robert Bosch Medical Center, Auerbachstrasse 110, 70376 Stuttgart, Germany; 2 Division of Cardiovascular Medicine, Radcliffe Department of Medicine, University of Oxford, John Radcliffe Hospital, Headley Way, Headington, Oxford, OX3 9DU UK

**Keywords:** Myocardial inflammation, Cardiovascular magnetic resonance, Gadolinium, Tissue characterization, Edema, Mapping

## Abstract

Several exogenous or endogenous factors can lead to inflammatory heart disease. Beside infectious myocarditis, other systemic inflammatory disorders such as sarcoidosis, systemic lupus erythematosus (SLE), systemic sclerosis (SSc), Churg-Strauss syndrome, and rheumatoid arthritis can affect the myocardium. Myocardial inflammation may have a major impact on the outcome of these patients, resulting in sudden cardiac death, severe arrhythmias, or end-stage heart failure. The current gold standard for definite confirmation of inflammatory heart disease is endomyocardial biopsy (EMB), but is invasive and suffers low sensitivity and specificity due to sampling errors. Thus, non-invasive methods for detecting the extent and changes over time of the inflammatory myocardial disease are needed. Cardiac magnetic resonance (CMR) is such a non-invasive method. We will describe and discuss different approaches for CMR assessment of inflammatory myocardial disease including early gadolinium enhancement (EGE), T2-weighted imaging, late gadolinium enhancement (LGE), the newer mapping proton relaxation techniques (T1 pre-contrast, T1 post-contrast, T2 mapping), and the hybrid PET/MRI technique.

## Introduction

Several exogenous or endogenous factors can lead to acute or chronic inflammatory heart disease. Beside infectious myocarditis, which is mostly viral in origin, other systemic inflammatory disorders such as sarcoidosis, systemic lupus erythematosus (SLE), systemic sclerosis (SSc), Churg-Strauss syndrome, and rheumatoid arthritis can affect the myocardium.

Myocardial inflammation may have major impact on the outcome of these patients, resulting in sudden cardiac death, severe arrhythmias, or end-stage heart failure [1••]. Currently, a definite diagnosis of inflammatory heart disease requires endomyocardial biopsy (EMB) [2], which is the only procedure that can directly assess the extent of myocardial inflammation and clarify the underlying pathophysiology and its triggers, potentially yielding to adequate treatment of these patients. However, it is invasive and suffers from several limitations including low sensitivity and specificity due to sampling errors. Thus, new non-invasive methods for detecting and following inflammatory myocardial disease are needed in addition to biopsy.

Cardiac magnetic resonance (CMR) is such a new non-invasive method, which has high spatial resolution, allows evaluation of morphology, function, and tissue characterization as a one stop shop, and works without any radiation. In this manuscript, we will describe and discuss different approaches for CMR assessment of inflammatory myocardial disease (see Table 1) including early gadolinium enhancement (EGE), T2-weighted imaging, late gadolinium enhancement (LGE), the newer mapping proton relaxation techniques (T1 pre-contrast, T1 post-contrast, T2 mapping), and the hybrid novel PET/MRI technique.

**Table 1:** How to approach myocardial inflammation by CMR

CMR technique	Pros	Cons
Early gadolinium enhancement (EGE)	Hyperemia	Image quality/artifacts Scarce validation against histology Mechanism unclear
T2-weighted images	Edema, free water	Image quality/artifacts Scarce validation against histology No prognostic data
Late gadolinium enhancement (LGE)	Scar/necrosis Best validation against histology Widely available/usually good quality Prognostic impact	Indicating irreversible damage but not specific for inflammation Only focal processes Correct inversion time important
T1 mapping native	Diffuse fibrosis Edema, free water No contrast agent Short acquisition protocol	Not clinical routine Variety of sequences Different normal ranges High T1 not specific
Extracellular volume (ECV) quantification	Expansion of extracellular space: edema, fibrosis, amyloid deposition	Not clinical routine Motion artifacts
T2 mapping	Edema, free water No contrast agent Short acquisition protocol	Not clinical routine Interindividual variability
PET/MR	Combining detection of inflammation (PET) with high-resolution tissue characterizing (MR)	Expensive Not widely available Radiation exposure

## CMR Techniques

### Early Gadolinium Enhancement (EGE)

Acute inflammation leads to hyperemia, and some reports describe an increased early uptake of gadolinium in these areas [3]. Thus, after contrast application, signal intensity is elevated in the first few minutes compared to skeletal muscle, which can be quantified on spin echo images. However, the exact mechanism of EGE is less clear and non-specific, and image quality can be significantly affected by motion artifacts due to irregular heart rhythms and breathing patterns. Hence, EGE alone is usually insufficient for the diagnosis of myocardial inflammation. It is most informative when positive on good-quality images in combination with other tissue characterization techniques, and while it can be used clinically, many centers tend to reserve EGE as a research tool.

### T2-Weighted Images

T2 is the time of transverse magnetization decay, which is prolonged in edematous, inflammatory myocardial tissue, appearing bright on T2-weighted images. Similar to EGE and using T2 dark-blood sequences, myocardial signal is indexed to skeletal muscle signal intensity. The underlying assumption to this approach is, that skeletal muscle is not influenced by inflammation, which seems quite problematic in acute viral or systemic inflammatory diseases.

On dark-blood T2-weighted imaging, the diagnosis of global myocardial edema is made when the ratio is >1.9 [4]. Unfortunately, image quality of this method depends heavily on a regular, normofrequent rhythm, and patient ability to breath-hold during the image acquisition. Furthermore, this method is prone to bright signal artifacts adjacent to the subendocardium due to poor dark-blood preparation of slow-flow blood within the LV cavity. Newer bright-blood T2-weighted sequences overcome some of these limitations, but were not shown to be superior to dark-blood T2-weighted imaging in the evaluation of global myocardial edema and myocarditis [5•, 6••].

### Late Gadolinium Enhancement

LGE images are acquired late (15–25 min) after application of gadolinium. At that time, contrast is washed out in normal myocardium (which is nulled), but in regions with enlarged extracellular/interstitial space due to acute necrosis, chronic scar, or fibrotic tissue, the contrast agent exhibit delayed washout, appearing hyperintense on the images [7]. Hence, in comparison to EGE and T2-weighted imaging, LGE typically reveals areas of irreversible damage, such as acute necrosis and chronic fibrosis. It has its strengths in detecting focal processes, whereas in diffuse processes this technique has its limitations. Nevertheless, LGE is the most frequently used and best established method for myocardial tissue characterization, useful not only in diagnosing non-ischemic cardiac diseases, but also in prognostication [1••, 8•, 9•]. Furthermore, in comparison to EGE and T2-weighted imaging, LGE is best validated against histological evidence of myocarditis [10, 11].

### “Two Out of Three Is Better than One?”

There is an ongoing debate about the best diagnostic approach to inflammatory heart disease using different CMR sequences. In 2009, some experts formulated an approach to detect myocardial inflammation using EGE, T2-weighted, and LGE imaging. Based on collective evidence available at that time, albeit small number studies, the diagnostic accuracy of acute myocarditis may be improved when two out of the three imaging techniques were positive, also known as the “Lake Louise criteria” [12]. This is sensible in principle, as increasing the number of measurements may increase the diagnostic yield for detecting disease, although EGE and T2-weighted techniques often require longer acquisition times and image quality prone to motion artifacts, making these challenging to apply especially in acutely ill patients who have tachyarrhythmia and difficulty breath-holding.

### T1 Mapping Pre-/post-contrast

Recently developed pre-contrast T1 quantitative mapping techniques represent a fundamental step forward in the non-invasive assessment of myocardial inflammation [13]. Despite saturation recovery and inversion recovery based T1 mapping techniques being developed since the 1980s [14, 15], important challenges impeded their clinical application [16]. It is only since a more robust and reproducible mapping sequence (e.g., the modified Look-Locker inversion recovery (MOLLI) sequence) [17] has become available that wider clinical applications have become possible. Technical advances allowing for shorter acquisitions and shorter breath-holds (such as the ShMOLLI technique) facilitate the implementation in acutely ill patients [18, 19•, 20].

Post-contrast T1 mapping techniques are also available; these are currently being used to assess for expansion of interstitial space/fibrosis (extracellular volume, ECV) and distinguish it from replacement fibrosis (such as scarred myocardium) [21, 22]. Using an extravascular gadolinium contrast medium, once an equilibrium distribution between blood and tissue is attained, the partition coefficient of the contrast can be derived using pre- and post-contrast T1 values of the blood and myocardium. Further methods such as equilibrium contrast CMR (EQ-CMR) [23] and dynamic equilibrium CMR [24] adjust for the hematocrit to derive the ECV.

The diagnostic accuracy of T1 mapping techniques can be hampered by motion artifacts due to inconsistent breath-holding or patient movements. To overcome these limitations, technical developments allowing for motion correction and co-registration are now available [25].

### T2 Mapping

Spin echo-based T2 mapping techniques are known to be prone to effects of cardiac motion and stimulated echoes [26]. T2 mapping techniques using T2-prepared steady-state free precession (T2p-SSFP) were initially developed for BOLD imaging [27]. Using a similar mapping scheme, Giri et al. [28], validated the technique and demonstrated its increased accuracy in detecting T2 variations across the myocardium compared not only to standard T2-weighted techniques but also to spin echo-based T2 mapping techniques. Further developments aiming specifically to address misregistration of the parametric maps are available using navigator gating and map registration, enabling a more robust-free breathing T2 mapping technique [29].

### T1/T2 Mapping in Myocardial Inflammation/Edema

Myocardial inflammation and edema are characterized by an increase in free water content, which prolongs both T2 and T1 relaxation times. Myocardial T1 and T2 mapping have been validated against microspheres to detect myocardial edema in experimental animal models [30••] and also in human clinical populations with superior diagnostic performance to conventional T2-weighted techniques [5•, 31]. Quantitative T1 and T2 mapping techniques are well suited to detect myocardial inflammation and edema, which are common final pathways of myocardial injury in a number of myocardial conditions, with potentially high sensitivity and ability to locate small areas of abnormality.

### PET/MRI

PET/MRI combines the strengths of both techniques. PET is a non-invasive imaging technique, which can detect metabolic active processes with high sensitivity: ^18^F-FDG PET cumulates in inflammatory regions due to the glycolytic activity of cells, which participate in inflammatory processes (neutrophils, monocytes/macrophages). Disadvantages of PET are radiation exposure and reduced spatial resolution, so small areas of inflammation might be missed. Hence, a combination with MRI (providing high spatial resolution and lack of radiation exposure) seems reasonable [32].

## Clinics

### Myocarditis

Viral myocarditis is the most common inflammatory heart disorder. Symptoms and clinical presentation may vary, making the diagnosis a challenge. Furthermore, the clinical course comprises a wide spectrum ranging from complete recovery to terminal heart disease or sudden cardiac death. Autopsy studies revealed that myocarditis is responsible for 5 to 20 % of sudden deaths in young adults [33], so getting the diagnosis right is of vital importance in this population.

CMR and its unique ability of non-invasive tissue characterization are extremely helpful in the work-up of suspected myocarditis. There are three stages of myocardial alterations in these patients: firstly, myocardial damage due to the trigger; secondly, myocardial inflammation by the host immune system; and thirdly, chronic myocardial inflammation, which may result in myocardial fibrosis, leading to remodeling, consequent ventricular dysfunction [34] and arrhythmic substrates.

**Fig. 1:**
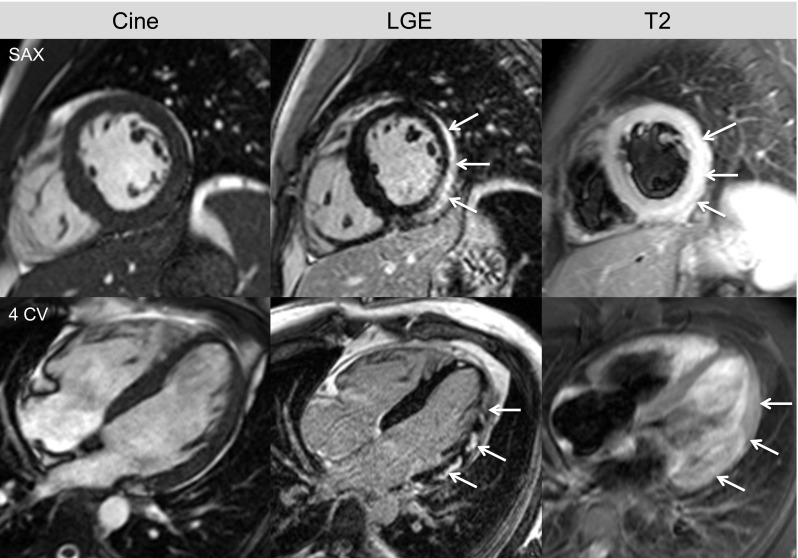
CMR of a 28-year-old male presenting with dyspnea and chest pain 5 days after flu-like symptoms. Due to ST elevation and elevated cardiac enzymes, the patient underwent coronary angiography where coronary stenosis could be excluded. Cine images reveal global preserved left ventricular ejection fraction (LVEF = 60 %) with discrete hypokinesia at the lateral wall. LGE is present in multiple locations (*white arrows*), suggestive of myocarditis. The acute nature of the disease is demonstrated by positive dark-blood T2W images displaying a clear bright signal in the lateral wall, in an area consistent with LGE findings. Note that on the correspondent T2-weighted images in the long-axis, these lesions are not well delineated. This patient received state of the art heart failure medication (betablocker, ARB); in the next few months, the initial extensive LGE lesions decreased in size (which could be observed at a subsequent CMR scan) and the patients complaints resolved completely

These alterations lead to acute myocardial necrosis and/or chronic scarring/fibrosis reflected by subepicardial or intramural regions of LGE, often patchy, and preferentially located in the posterolateral wall [10], see Fig. 1.

In the setting of viral myocarditis, CMR studies with LGE are being performed for more than 10 years, first looking at the diagnostic performance of the technique, followed by prognostic data. To date, there is clear evidence that positive LGE is associated with an adverse outcome: patients with LGE are at increased risk of suffering adverse cardiac events. Grün et al. demonstrated in more than 220 patients with biopsy-proven viral myocarditis that the presence of LGE is the best independent predictor of all-cause mortality and of cardiac mortality [1••]. Furthermore, patients with no LGE have an excellent prognosis. This was confirmed by Schumm and Greulich et al. in a large study with more than 400 patients with suspected myocarditis (“all comers”): patients who had a CMR scan without pathological findings (normal left ventricular ejection fraction, normal left ventricular enddiastolic volume, no LGE) had a good prognosis, independent of their clinical symptoms and other findings [9•]. Nevertheless, we have to keep in mind that not all LGE-positive patients will suffer an event, but they are at an increased risk to do so.

Despite the limitations of EGE and T2-weighted imaging, these techniques may be useful in supporting the acute nature of the inflammatory process when images are diagnostic. Friedrich et al. recently published data on 35 patients with suspected acute myocarditis, in whom the overall diagnostic accuracy of “T2 and/or LGE” was significantly better than LGE alone. For patients in whom a high positive likelihood ratio is needed, the full Lake Louise criteria may be useful [35]. However, as described above, these techniques can be challenging to apply in the practical clinical setting, and novel quantitative mapping techniques may circumvent many of the recognized technical and methodological limitations.

### T1 Mapping and ECV

Recently, mapping techniques have been shown to have great utility in this disease owing to its sensitivity to detecting myocardial water and pixel-wise quantification. Ferreira and Piechnik et al. [6••] first demonstrated that native T1 mapping using the ShMOLLI technique [18, 36••] is a superior diagnostic technique (ROC area-under-the-curve 0.95) compared to conventional T2-weighted imaging, with an equivalent performance to LGE. Not only can native T1 mapping be used as a novel diagnostic criterion like the Lake Louise criteria, with superior sensitivity to T2-weighted and LGE imaging, but native T1 maps can also display the typical non-ischemic patterns in acute myocarditis without the need for gadolinium contrast agents [37]. In addition, T1 mapping offers a significant incremental diagnostic value, detecting additional areas of myocardial involvement beyond T2-weighted and LGE imaging and identified extra (30 %) cases when at least one of these conventional methods failed to identify abnormalities [37].

Compared to the diagnostic performance of some of the Lake Louise criteria, native T1 mapping, as a method for imaging edema, may replace dark-blood T2-weighted imaging either as a single criterion or in combination with LGE. ShMOLLI T1 mapping offers a number of advantages to conventional dark-blood T2-weighted imaging such as for cases in which tachyarrhythmia, patient inability to perform long breath-hold or skeletal muscle inflammation lead to non-diagnostic or false-negative images. The added ability of T1 mapping to demonstrate non-ischemic patterns of myocardial injury using incremental thresholds means that T1 mapping can effectively act as a method for both edema and LGE pattern imaging. In the future, it may be possible to perform a gadolinium-free CMR protocol using cine and T1 mapping, with T1 mapping as a single criterion demonstrating around 90 % in sensitivity, specificity, accuracy, PPV, and NPV [37].

Luetkens et al. [38] compared the diagnostic performance of the Lake Louise criteria, native T1 relaxation times and ECV at 3 Tesla and found that native T1 mapping significantly outperformed the other techniques tested (ROC area under the curve 0.94), with a high diagnostic performance (sensitivity 92 %, specificity 91 %, diagnostic accuracy 91 %), consistent with the results by Ferreira et al. [6••, 37]. The specificity of T1 mapping was significantly higher with native T1 mapping than that of the combined Lake Louise criteria (sensitivity, 92 %; specificity, 80 %; diagnostic accuracy, 85 %).

ECV quantification has utility in acute to subacute, severe myocarditis, as shown by Radunski et al. [39], who assessed the diagnostic performance of T2, T1, and extracellular volume (ECV) quantification as novel quantitative tissue markers compared to the Lake Louise criteria. ECV quantification with LGE imaging significantly improved the diagnostic accuracy of CMR compared with the Lake Louise criteria, in patients being evaluated 1–7 weeks after the index presentation.

### T2 Mapping

T2 mapping techniques have been shown to allow detection of myocardial edema beyond standard techniques such as wall motion abnormalities, T2-weighted, or LGE in a variety of cardiac pathologies including myocarditis. As shown by Thavendiranathan et al. [31], T2 mapping has a sensitivity and specificity of 94 and 97 %, respectively, in detecting acute inflammatory diseases such as myocarditis and Takotsubo cardiomyopathy. T2 mapping had also shown some utility in detecting severe myocarditis up to 7 weeks after presentation [39].

Overall, while quantitative mapping techniques hold promise to diagnose myocarditis, establishing which method has the best diagnostic accuracy is still challenging and requires further evidence, such as larger studies and multicenter trials.

### Sarcoid Myocarditis

**Fig. 2:**
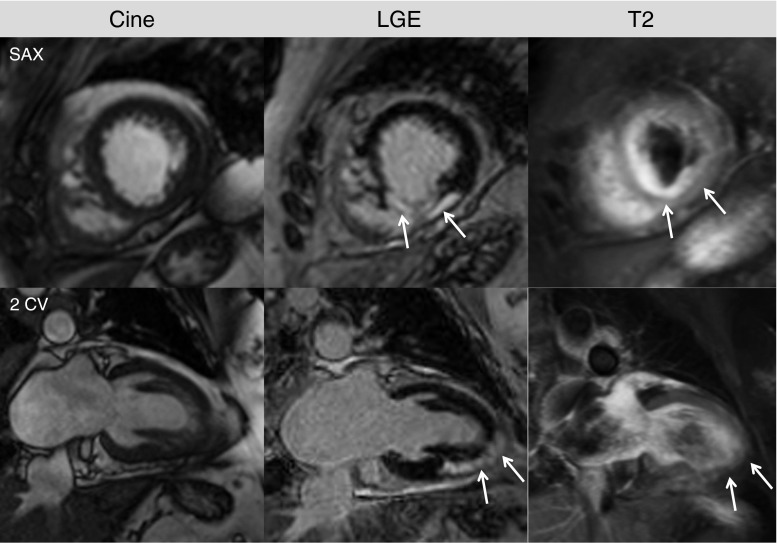
A 61-year-old female presenting with chest pain and elevated cardiac enzymes. Coronary artery disease (CAD) was ruled out by coronary angiography. **a** Cine images showed a slightly diminished left ventricular ejection fraction (LVEF = 53 %). **b** LGE images revealed an almost transmural LGE inferoseptal from midventricular to the apex, from epicardial origin. This pattern could indicate cardiac manifestation of a systemic disease such as sarcoid. **c** T2-weighted images demonstrate hyperintense regions (edema) in the inferoseptal wall (*white arrows*), corresponding to LGE. Biopsies were taken, which confirmed the diagnosis of sarcoidosis, and steroid therapy was initiated

Sarcoidosis is a systemic granulomatous inflammatory disease with unknown etiology that can affect the heart additionally, or isolated. In case of cardiac involvement, typical manifestations include arrhythmias, heart failure and/or sudden cardiac death, which is a frequent cause of death in these patients. Prevalence of cardiac involvement ranges from 20 to 30 % [40], depending on the patient population. As described above, the gold standard endomyocardial biopsy has a low sensitivity, underlining the need of alternative (non-invasive) tools for diagnosis and management. In line with the results of Patel et al. [41], Greulich et al. could clearly demonstrate in a cohort of 155 patients with suspected cardiac sarcoidosis that the presence of LGE is correlated with major adverse advents such as ventricular tachycardia and sudden cardiac death [8•]. Thus, CMR screening for cardiac involvement in patients with sarcoidosis is now available in the clinical routine. Figure 2 illustrates a typical patient example, in which CMR revealed cardiac involvement of sarcoid disease.

**Fig. 3:**
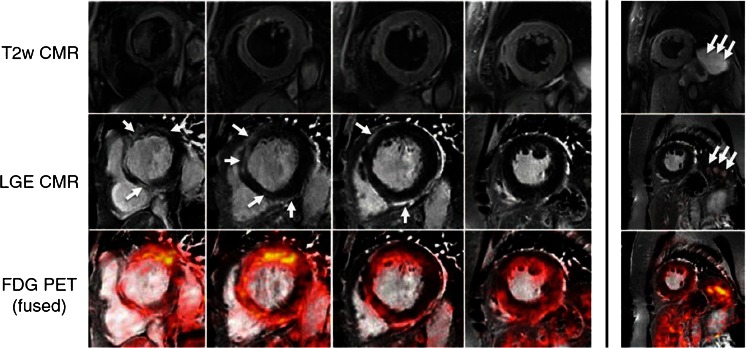
A 72-year-old woman was referred with increasing shortness of breath and intermittent chest pain. Echocardiography revealed a diminished left ventricular ejection fraction (35 %); significant CAD could be ruled out by coronary angiography. Biopsy of an enlarged scalene lymph node showed non-caseating granulomas, so cardiac sarcoid was considered, and a hybrid PET/MRI was scheduled. In the *upper row* T2-weighted images are displayed in cine short axis views. *Middle row* shows the concomitant LGE images; the presence of LGE is indicated by the *white arrows*. In the *bottom row*, there are fused images (MRI and PET): note that PET images show an increased uptake in the border zones of the LGE, suggesting that the process of inflammation affects more regions than displayed solely by LGE. *Far right*, CMR images showing splenic nodules with enhancement on both LGE sequences and by PET. *Reprinted with permission from LWW* [42]

PET/MRI is a promising approach in patients with known or suspected cardiac sarcoidosis to detect inflammation with high sensitivity (active cardiac sarcoidosis), as well as fibrosis by LGE (chronic cardiac sarcoidosis), which may have implications for diagnosis and clinical follow-up [42, 43]. First results are convincing (see Fig. 3), but prospective studies are needed. In addition, PET imaging is expensive and not widely available, yet. Furthermore, preparation for PET (12–18-h fasting to suppress physiologic glucose metabolism by the heart) and radiation exposure have to be considered in these (often young) patients. On the other hand, due to the wide implication of possible cardiac involvement, detailed diagnostic testing may be warranted.

### Collagenosis: Systemic Lupus Erythematosus (SLE) and Systemic Sclerosis (SSc)

The group of collagenosis consists of a heterogenous group of systemic diseases affecting connective tissue and is characterized by necrosis or vasculitis.

### Systemic Lupus Erythematosus

This disease affects predominantly females, with cardiac involvement reported in 60–70 % of the patients. These patients are exposed to an increased risk of coronary atherosclerosis, which may result in myocardial infarction. Beside of atherosclerosis, myocarditis and pericardial effusion could be observed, justifying the use of non-invasive imaging techniques. The prevalence of LGE in these patients ranges from low to high in the literature. This might be due to the fact that there is a wide spectrum of disease activity in these patients. Nevertheless, a study from Mavrogeni et al. [44], which compared 50 patients with suspected infective myocarditis and 25 active SLE patients to 20 controls revealed that LGE was positive only in the minority of SLE-active patients, underlining the need of additional CMR techniques, which can detect inflammatory processes.

#### T1 Mapping and ECV

Native T1 mapping and ECV quantification were able to detect subclinical myocardial involvement in patients with SLE who were asymptomatic and had normal LV indices [45•]. Compared to normal controls, both native myocardial T1 and extracellular volume fraction (26 ± 5 % vs. 30 ± 6 %) were significantly increased in patients with SLE, both of which also correlated with reduced longitudinal strain. Native myocardial T1 values showed the greatest concordance with the presence of clinical diagnosis of SLE compared to other CMR markers such as myocardial function, perfusion, and LGE. Thus, T1 mapping is able to demonstrate subclinical myocardial involvement in systemic diseases otherwise not apparent on conventional cardiac diagnostic tests.

### Systemic Sclerosis

In contrast to SLE, the role of SSc in the pathogenesis of coronary atherosclerosis is not as well defined: data range from elevated risk to risk equal to the general population. There is consensus about potential cardiac involvement, which can manifest as myocardial damage, disturbances of the conduction system, pericardial disease, or alterations of the valves. Aside from that, secondary cardiac complications may occur due to extracardiac manifestations of SSc: e.g., secondary pulmonary hypertension due to sclerotic pulmonary alterations.

A recent study [46] revealed that myocarditis is a common finding in SSc patients with recent-onset cardiac involvement, presenting with new onset of clinical symptoms (heart failure, chest pain, palpitations) and elevated cardiac enzymes: six out of seven patients showed LGE; all had evidence of acute or chronic myocarditis by histology. Only in two out of these six LGE positive patients, T2 hyperintensity (edema) was present. Despite the initiation of immunosuppressive treatment, two out of seven patients suffered SCD. This study underscores, that, if cardiac involvement is present, LGE may be a good prognosticator, and these patients may require a closer observation or maybe even a more aggressive treatment to decrease the likelihood of adverse cardiovascular events, although larger studies are needed. The combination of repeated ischemia/vasoconstriction and inflammation may result in diffuse interstitial fibrosis, which was reported in endomyocardial biopsies of SSc patients [47•].

### T1 Mapping and ECV

**Fig. 4:**
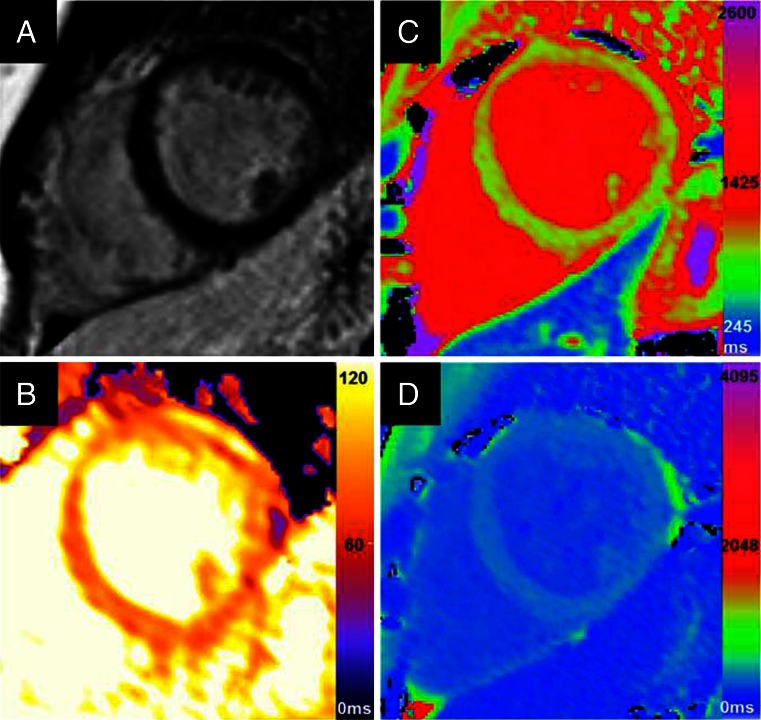
Cardiac magnetic resonance images from a 48-year-old patient with systemic sclerosis (SSc), with a normal left ventricular (LV) ejection fraction of 63 %. **a** LGE imaging with no focal areas of enhancement. **b** T2 map. **c** Native T1 map (acquired using ShMOLLI at 1.5 T) showed significantly elevated average LV myocardial T1 values of 1009 ± 47 ms (normal 962 ± 25 ms). **d** Post-contrast T1 map acquired at 15 min after gadolinium administration. Average post-contrast T1 values were 479 ± 18 ms. Extracellular volume (ECV) quantification using pre- and post-contrast T1 mapping and adjusting for the hematocrit demonstrated a significant expansion of interstitial space at 40 % (normal 27 ± 3 %). *Images courtesy Dr. Ntobeko Ntusi*, *University of Oxford*

Three groups of investigators have been able to detect early subclinical myocardial involvement in systemic sclerosis using T1 mapping and ECV quantification when there is no apparent cardiac disease. Ntusi et al. [48] found that in patients with SSc without overt cardiovascular disease, native T1 values were significantly elevated, detecting large areas of myocardial abnormality (median 52 vs. 3 % in controls) and expansion of ECV (35.4 ± 4.8 vs. 27.6 ± 2.5 %), likely resulting from a combination of low-grade inflammation and diffuse myocardial fibrosis (see Fig. 4). Native T1 and ECV were correlated with disease activity and severity, and ECV inversely correlated with subclinical myocardial dysfunction as reflected in impairment in the peak systolic circumferential strain and peak diastolic strain rate despite normal biventricular size and systolic function. Thuny et al. [49] reported similar findings in patients with SSc, normal LV systolic function, and no LGE, who had significantly increased ECV (median 30.0 vs. 26.8 % in controls), which correlated with left atrial volume and diastolic dysfunction. Barison et al. [50] also demonstrated ECV expansion in asymptomatic SSc patients with normal LV systolic function and without LGE, both in the myocardium (30 ± 4 % vs. 28 ± 4 % in controls) and in skeletal muscle (23 ± 6 % vs. 18 ± 4 %). T1 mapping and ECV quantification can detect subclinical myocardial disease in this condition and may identify patients with early myocardial involvement, with potential to treat early before permanent irreversible injury occurs.

### Churg-Strauss Syndrome

The vasculitides encompass a wide spectrum of diseases with different entities. Aside from large-vessel vasculitis (e.g., Takayasu, Kawasaki), vasculitis of the small vessels (EGPA = eosinophilic granulomatosis with polyangiitis, also known as Churg-Strauss syndrome) has gained attention, showing some impressive cardiac manifestation leading to impaired left ventricular function. Interestingly, LGE images show a subendocardial and also intramural pattern, sometimes resembling ischemic heart disease, but typically not related to a coronary territory, representing destructed coronary capillaries due to the inflammation process. More recent advances in characterizing inflammation by mapping sequences seem promising, but there is no published data yet.

### Rheumatoid Arthritis (RA)

Patients with rheumatoid arthritis (RA) suffer accelerated atherosclerosis. The prevalence of myocardial tissue involvement is very variable in the literature, due to different inclusion criteria and patient population.

Ntusi et al. [51] compared 39 RA patients (28 female, mean age 50 ± 12 years, diagnosed with RA for a median of 7 years, mean DAS28-CRP score 3.3 ± 1.3) to 39 matched controls, all without previously known cardiovascular disease using CMR at 1.5 T, including cine, T2-weighted, native T1 mapping, ECV quantification, and LGE imaging. RA patients had larger areas of focal myocardial edema on T2-weighted imaging (median 10 vs. 0 % LV involvement). Forty-six percent had LGE, of which 28 % showed a midwall pattern and 13 % patchy enhancement; two patients (5 %) revealed previously unknown myocardial infarction, subsequently confirmed to have a significant CAD on angiography. Overall, however, the LGE burden was small (3.7 ± 0.4 % of total LV mass).

### T1 Mapping and ECV

Further, the utility of T1 mapping and ECV has been shown in patients with RA but no prior history of cardiovascular disease and normal LV indices [51]. Compared to normal controls, native T1 and ECV (30.3 ± 3.4 % vs. 27.9 ± 2.0 %) were significantly elevated in these patients, regardless of the presence of LGE, and both correlated with impaired peak systolic and diastolic strain and RA disease activity. In RA patients with apparently normal hearts, subclinical myocardial involvement can be detected using conventional and mapping CMR techniques, which show focal myocardial fibrosis, inflammation, and expansion of extracellular space, likely reflecting a degree of diffuse myocardial fibrosis in this systemic disease. T1 mapping and ECV quantification may provide incremental value as novel biomarkers for early disease detection, monitoring, and determining response to therapy in systemic inflammatory diseases.

## Conclusion

In summary, CMR techniques are able to provide detailed information about myocardial inflammation. LGE is the most validated tool for diagnosis and prognosis, but has limitations in detecting acute inflammatory processes without scar or fibrosis. EGE and T2-weighted imaging may be useful in increasing the diagnostic accuracy of acute inflammatory myocardial processes in selected cases but can be challenging to apply in the practical clinical setting due to recognized technical and methodological limitations. Novel quantitative mapping techniques seem very promising, as they can detect edema as well as fibrosis while circumventing some of the challenges of conventional CMR techniques, allowing a more holistic approach to the different stages of inflammation. Prospective multicenter studies are warranted to clarify the future role of their clinical utility.
